# Efficacy and safety of core-needle biopsy in initially detected thyroid nodules via propensity score analysis

**DOI:** 10.1038/s41598-017-07924-z

**Published:** 2017-08-15

**Authors:** Chong Hyun Suh, Jung Hwan Baek, Young Jun Choi, Tae Yong Kim, Tae Yon Sung, Dong Eun Song, Jeong Hyun Lee

**Affiliations:** 1 Department of Radiology and Research Institute of Radiology, University of Ulsan College of Medicine, Asan Medical Center, 86 Asanbyeongwon-Gil, Songpa-Gu, Seoul 138-736 Republic of Korea; 2 Department of Radiology, Namwon Medical Center, 365, Chungjeong-Ro, Namwon-Si, Jeollabuk-Do 590-702 Republic of Korea; 3 Department of Endocrinology and Metabolism, University of Ulsan College of Medicine, Asan Medical Center, 86 Asanbyeongwon-Gil, Songpa-Gu, Seoul 138-736 Republic of Korea; 4 Department of Surgery, University of Ulsan College of Medicine, Asan Medical Center, 86 Asanbyeongwon-Gil, Songpa-Gu, Seoul 138-736 Republic of Korea; 5 Department of Pathology, University of Ulsan College of Medicine, Asan Medical Center, 86 Asanbyeongwon-Gil, Songpa-Gu, Seoul 138-736 Republic of Korea

## Abstract

We compared the efficacy and complications of core-needle biopsy (CNB) with those of fine-needle aspiration (FNA) in a large population of patients with initially detected thyroid nodules via a propensity score analysis. Outpatients with initially detected thyroid nodules, who had undergone CNB or FNA between January 2013 and December 2013, were selected. This study included 4,822 thyroid nodules from 4,553 consecutive patients. Adjustments for significant differences in patients’ baseline characteristics were facilitated via propensity score analysis. Subgroup analyses were performed according to nodule sizes ≥ 1 cm. The non-diagnostic result rate, malignancy rate, complication rate, and diagnostic accuracy were compared. A 1:1 matching of 1,615 patients yielded no significant differences between two groups for any covariate. The non-diagnostic result rate was significantly lower in the core-needle biopsy group than in the fine-needle aspiration group (5.2% vs. 12.1%), while the malignancy rate (23.7% vs. 11.8%) and sensitivity (75.9% vs. 55.6%) were significantly higher. However, the specificities were similar (100% and 99.9%, respectively). Propensity score and subgroup analyses showed similar results. The complication rate was similar between groups in matched cohorts. CNB is a promising and safe diagnostic tool for patients with initially detected thyroid nodules.

## Introduction

Ultrasonography (US) guided, fine-needle aspiration (FNA) is considered to be a standard diagnostic tool for thyroid nodules, and is recommended by current practice guidelines^1^. The non-diagnostic result rate on FNA has been reported to range from 10 to 33.6%^2–5^. Patients with non-diagnostic result typically require a repeat biopsy. In addition, repeated non-diagnostic result can also result in unnecessary or diagnostic surgery^2^.

Core-needle biopsy (CNB), as an alternative to FNA, has been introduced as a procedure for thyroid nodules with previously non-diagnostic or indeterminate results. CNB is considered to be safe and well tolerated with a low incidence of complications^6–12^. Several recent studies demonstrated that CNB can effectively reduce the non-diagnostic result rate^11, 13–16^ and minimize unnecessary and/or diagnostic surgery^10, 11, 14^ for patients with thyroid nodules initially showing non-diagnostic or indeterminate results via FNA^17^. A recent small population pilot study (31 patients) reported that first-line use of CNB was more effective for suspicious thyroid nodules on US, compared to FNA^18^.

Despite the advantages of CNB for thyroid nodules with previously non-diagnostic or indeterminate results, the efficacy and safety of CNB for initially detected thyroid nodules remain unclear^19^. Therefore, we compared the efficacy and complications of CNB with those of FNA in a large population of patients with initially detected thyroid nodules via a propensity score analysis.

## Methods

### Study population

The protocol of this observational study was approved by the institutional review board of Asan Medical Center, a tertiary referral center, which waived the requirements for informed written consent for use of these data. All methods were performed in accordance with the relevant guidelines and regulations.

**Figure 1:**
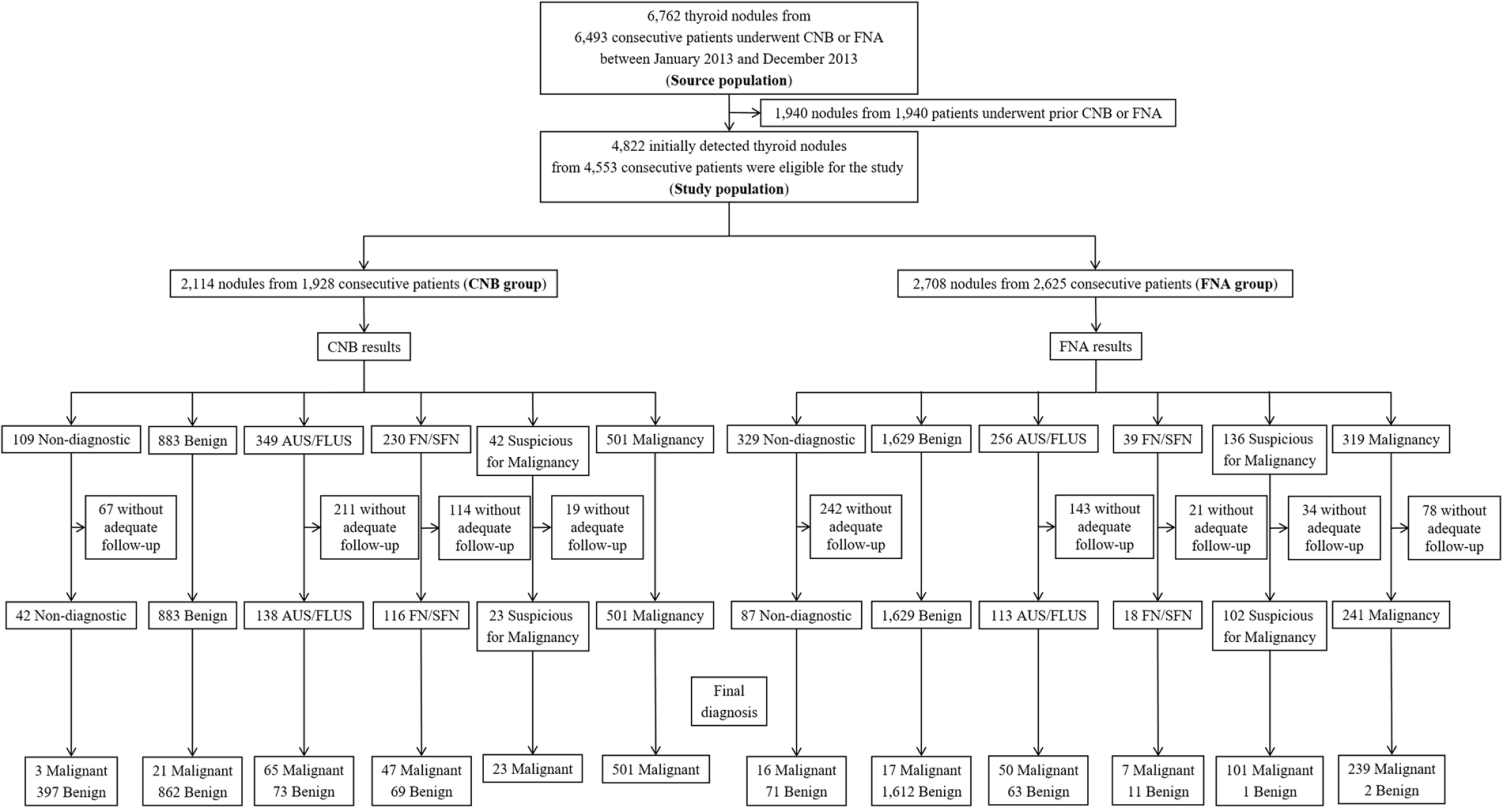
Patient flow and study outcomes in the study patients. Numbers represent the number of thyroid nodules. CNB = core-needle biopsy, FNA = fine-needle aspiration, AUS = atypia of undetermined significance, FLUS = follicular lesion of undetermined significance, FN = follicular neoplasm, SFN = suspicious for a follicular neoplasm, US = ultrasound.

The study population was obtained from a historical cohort of 6,762 thyroid nodules from 6,493 consecutive patients who underwent CNB or FNA between January 2013 and December 2013 at Asan Medical Center, an academic, tertiary referral hospital located in South Korea. Patients who had previously undergone CNB or FNA were excluded (n = 1,940). Finally, a total of 4,822 cases of initially detected thyroid nodules from 4,553 patients were included in this study: 2,114 nodules from 1,928 patients who had undergone CNB (CNB group) and 2,708 nodules from 2,625 patients who had undergone FNA (FNA group). Figure 1 shows the flowchart for CNB and FNA patient inclusion.

As a reference standard, malignancy was diagnosed after surgery or after CNB. Benign nodules were diagnosed accordingly: after surgery; after CNB; after at least two sets of benign findings on FNA on different occasions; or after benign cytology findings on FNA with the nodule size remaining stable after 1 year^20^.

### Analysis of US findings

US examinations were performed using one of the three US systems, i.e. an iU22 or an HDI-5000 unit (Philips Healthcare, Bothell, WA), or an EUB-7500 unit (Hitachi Medical Systems, Tokyo, Japan). Each system was equipped with a linear, high-frequency probe (5–14 MHz). All US-guided procedures were performed by radiologists under the supervision of two faculty radiologists (J.H.B. and J.H.L., with 19 and 14 years of clinical experience, respectively, in performing and evaluating thyroid US). US-guided CNB and FNA procedures for thyroid nodules were performed according to current practice guidelines^21^. US-guided CNBs were performed under local anesthesia with 1% lidocaine using a disposable 1.1- or 1.6-cm excursion, 18-gauge, double-action, spring-activated needle (TSK Ace-cut; Create Medic, Yokohama, Japan)^11, 14, 15, 20^. Before insertion of the core needle, the vessels along their approach route were evaluated by power Doppler US in order to prevent hemorrhage. Using a freehand technique, the core needle was advanced from the isthmus of the thyroid toward the nodule^20^. After the needle tip had been advanced towards the edge of the nodule, the distance of fire (1.1 or 1.6 cm) was measured before sequential firing of the stylet and cutting cannula of the needle^20^. Several reports suggested that CNB may be beneficial for thyroid nodules with certain characteristics of nodules such as calcification or predominantly cystic component^22, 23^. On the basis of these evidences, we performed CNB for heavily calcified nodules and predominantly cystic nodules for which FNA may be less effective. Additionally, whether to perform CNB or FNA was determined according to the referring physicians’ preference.

US-guided FNAs were routinely performed using a 23-gauge needle. Direct smears were made in all cases, and all smears were immediately fixed with alcohol after the FNA procedure and were stained with Papanicolaou^15^. The number of needle passes was determined by the operator during the procedure, and a maximum of four needle passes were permitted for each nodule. Additional FNAs were recommended in the case of incomplete visual assessment results. Maximum number of FNAs performed during a single session was three.

The adequacy of the procedure was monitored via real-time US, and the adequacy of the tissue core was assessed by visual inspection^20^. An additional CNB was performed when targeting of the lesion was considered inaccurate. Maximum number of CNBs performed during a single session was two. Each patient was observed after firm local compression of the biopsy site for 10–20 minutes following the biopsy. If patients complained of pain or neck swelling, a repeat US examination was performed in order to evaluate possible complications.

### Histopathologic analysis of CNB specimens and cytopathologic analysis of FNA

All CNB specimens and FNA cytological analyses were reviewed by a thyroid cytopathologist (D.E.S., with 11 years of clinical experience in thyroid cytopathology). FNA cytology diagnoses were categorized into six categories according to the Bethesda System for Reporting Thyroid Cytopathology^15, 24^. Although standardization of CNB diagnostic criteria for thyroid nodules had yet to be established during our study, the histologic results of CNB were categorized into the six categories of the Bethesda System (Supplementary materials)^11, 14, 15, 24, 25^.

### Image review

The US images were reviewed independently by two radiologists (C.H.S. and J.H.B. with 5 and 19 years of clinical experience, respectively, in performing and evaluating thyroid US), neither of whom had any information regarding the patient’s clinical history, previous imaging results, or histologic results. Discrepancies between the US findings of the two investigators were resolved by consensus. The US findings for the nodules were evaluated for the following features: internal composition (solid, predominantly solid, predominantly cystic, or cystic), shapes (ovoid to round, taller than wide, or irregular), margins (smooth, spiculated, or ill-defined), echogenicity (isoechoic, hypoechoic, markedly hypoechoic, or hyperechoic), and the presence of microcalcifications, macrocalcifications, or rim calcifications^20, 26, 27^.

### Statistical analysis

A comparison of study outcomes included non-diagnostic result rate, malignancy rate, complication rate, and diagnostic accuracy. Major and minor complications were defined by the Society of Interventional Radiology^28, 29^. A major complication was defined as either a potentially life-threatening event requiring clinical treatment, or that which may lead to substantial morbidity or disability, or result in lengthened hospitalization^28, 29^. All other complications were considered as minor. The diagnostic criteria for malignancy were defined as Bethesda category 6 (malignancy). The diagnostic accuracy of CNB included its sensitivity and specificity for a diagnosis of malignancy.

It was anticipated that the CNB and FNA groups would differ substantially with respect to preprocedural characteristics. Therefore, we collected information on baseline variables that were available in both CNB and FNA registries to facilitate adjusted comparisons. In order to limit the effects of selection bias and potentially confounding variables in this observational study, we performed adjustments for significant differences evident in patient baseline characteristics via the use of propensity score matching and inverse probability weighting^30–32^. Propensity scores estimating probability, on the basis of patient characteristics, were developed with the use of a logistic regression model in the adjustment of between-group differences evident in patient baseline characteristics. After all propensity score matching was performed, we compared the baseline variables between the two groups. Distribution of propensity scores was evaluated via procedural modality examining the sufficient overlap between groups in ensuring comparability^31^. In assessing the adequate level of calibration, c-statistic and Hosmer and Lemeshow Goodness-of-Fit tests were performed. Inverse probability weighting, which is a propensity score-based technique that can be used to compensate for imbalances in study groups, was also performed^32^. Additionally, subgroup analyses were performed according to nodule sizes ≥ 1 cm. Significance was defined as *p* < 0.05. All statistical analyses were performed using SAS 9.4 (SAS Institute, Cary, NC).

## Results

### Characteristics of the study population

All patients tolerated the CNB and FNA procedures. The baseline characteristics of the study patients, according to the procedure, are shown in Table 1. In the CNB group, these patients included 446 men and 1,668 women with a mean age of 53.4 ± 12.6 years (mean ± SD). The mean size of the 2,114 nodules was 1.68 ± 1.23 cm, with 1,396 nodules (66.0%) being ≥ 1.0 cm. Of these patients: 860 underwent CNB because their nodules had suspicious features seen on US; 125 had heavily calcified nodules; and 72 had predominantly cystic nodules. The remaining 1,057 patients underwent CNB due to their referring physician’s preference. In the FNA group, these patients included 639 men and 2,069 women with a mean age of 53.9 ± 11.8 years (mean ± SD). The mean size of the 2,708 nodules was 1.24 ± 0.82 cm, with 1,474 nodules (54.5%) being ≥ 1.0 cm. Significant differences were evident between the two groups in terms of nodule composition, margin, echogenicity, and calcification. The frequency of suspicious US characteristics including spiculated margins, marked hypoechoicity, and micro/macrocalcifications in the CNB group was significantly higher than that in the FNA group^26^. The mean patient follow-up periods were 20.8 ± 13.7 months in the CNB group and 20.6 ± 13.9 months in the FNA group.

**Table 1:** Baseline Characteristics of the Pooled and Matched Cohorts.

Characteristics	Pooled Cohorts				Matched Cohorts			
	CNB (n = 2,114)	FNA (n = 2,708)	P value	Standardized Difference	CNB (n = 1,615)	FNA (n = 1,615)	P value	Standardized Difference
Clinical factors								
Age (years)	53.4 ± 12.6	53.9 ± 11.8	0.169	0.0398	53.4 ± 12.7	53.2 ± 12.1	0.815	−0.0082
Sex (M:F)	446:1,668	639:2,069	0.040	0.06	347:1268	334:1281	0.575	−0.0197
US Characteristics								
Nodule size (cm)	1.68 ± 1.23	1.24 ± 0.82	<0.001	−0.4293	1.44 ± 0.97	1.39 ± 0.94	0.147	−0.051
<1.0 cm	718 (34.0%)	1,233 (45.5%)	<0.001	0.2388	625 (38.7%)	643 (39.8%)	0.517	0.0228
≥1.0 cm	1,396 (66.0%)	1,474 (54.5%)			990 (51.3%)	972 (60.2%)		
Composition, n (%)			<0.001	0.3108			0.745	0.0391
Solid	1,722 (81.5%)	1,870 (69.1%)			1278 (79.1%)	1295 (80.2%)		
Predominantly solid	312 (14.8%)	584 (21.6%)			265 (16.4%)	244 (15.1%)		
Predominantly cystic	72 (3.4%)	217 (8%)			64 (4.0%)	69 (4.3%)		
Cystic	8 (0.4%)	37 (1.4%)			8 (0.4%)	7 (0.4%)		
Shape, n (%)			0.481	0.035			0.695	0.03
Ovoid to round	1,714 (81.1%)	2,229 (82.3%)			1309 (81.0%)	1290 (79.9%)		
Taller than wide	193 (9.1%)	223 (8.2%)			150 (9.3%)	161 (10.0%)		
Irregular	207 (9.8%)	256 (9.5%)			156 (9.6%0	164 (10.1%)		
Margin, n (%)			<0.001	0.1773			0.690	0.0303
Smooth	1,131 (53.5%)	1,627 (60.1%)			887 (54.9%)	864 (53.5%)		
Spiculated	257 (12.2%)	202 (7.5%)			166 (10.3%)	176 (10.9%)		
Ill-defined	726 (34.3%)	879 (32.5%)			562 (34.8%)	575 (45.6%)		
Echogenicity, n (%)			<0.001	0.2801			0.736	0.0397
Isoechoic	825 (39.0%)	1,328 (49.0%)			193 (12.0%)	203 (12.6%)		
Hypoechoic	956 (45.2%)	1,116 (41.2%)			702 (43.5%)	702 (43.5%)		
Markedly hypoechoic	318 (15.0%)	218 (8.1%)			705 (43.7%)	700(43.3%)		
Hyperechoic	15 (0.7%)	46 (1.7%)			15 (0.9%)	10 (0.6%)		
Calcifications, n (%)			<0.001	0.4002			0.699	0.0421
None	1,365 (64.6%)	2,189 (80.8%)			163 (10.1%)	178 (11.0%)		
Microcalcifications	217 (10.3%)	219 (8.1%)			241 (14.9%)	236 (14.6%)		
Macrocalcifications	407 (19.3%)	248 (9.2%)			61 (3.8%)	52 (3.2%)		
Rim calcifications	125 (5.9%)	52 (1.9%)			1150 (71.2%)	1149 (71.2%)		

### Characteristics of the patients matched for propensity scores

Propensity score matching was performed for the entire population. A total of 1,615 patients who underwent CNB were matched 1:1 with patients who underwent FNA for age, sex, US characteristics that included nodule size, composition, shape, margin, echogenicity, and calcifications by propensity score matching. The logistic model including the 8 variables yielded a c-statistic of 0.7243. Hosmer and Lemeshow Goodness-of-Fit test showed that the propensity score model had an adequate level of calibration (*p* = 0.164). In matched cohorts, there were no longer any significant difference between the CNB group and the FNA group for any covariate (Table 1).

### Outcomes for the pooled cohorts and the matched cohorts

The results and final diagnoses of the CNB and FNA groups are summarized in Table 2. Table 3 shows the study outcome according to the procedure modality in pooled cohorts and the matched cohorts. Non-diagnostic result rate was significantly lower in the CNB group (5.2%; 109 of 2,114 nodules) than in the FNA group (12.1%; 329 of 2,708 nodules; *p* < 0.001). Malignancy rate was significantly higher in the CNB group (23.7%; 501 of 2,114 nodules) than in FNA group (11.8%; 319 of 2,708 nodules; *p* < 0.001). The complication rate was slightly higher in the CNB group (0.33%; 7 of 2,114 nodules) than in the FNA group (0.074%; 2 of 2,708 nodules; *p* = 0.048). None of the included patients experienced any major complications associated with intervention or hospitalization. Eight patients developed a hematoma after the procedure, but resolution of the hematoma occurred following compression and rest for 1 hour. One patient developed a vasovagal reaction.

**Table 2:** CNB and FNA results and final diagnosis for initially detected thyroid nodules.

	Total CNB (n = 2,114)	Final diagnosis (n = 1,703)		Total FNA (n = 2,708)	Final diagnosis (n = 2,190)	
		Benign (n = 1,043)	Malignant (n = 660)		Benign (n = 1,760)	Malignant (n = 430)
Bethesda category 1 (Non-diagnostic)	109 (5.2%)	39 (3.7%)	3 (0.5%)	329 (12.1%)	71 (4.0%)	16 (3.7%)
Bethesda category 2 (Benign)	883 (41.8%)	862 (82.6%)	21 (3.2%)	1,629 (60.2%)	1,612 (91.6%)	17 (4.0%)
Bethesda category 3 (AUS or FLUS)	349 (16.5%)	73 (7.0%)	65 (9.9%)	256 (9.5%)	63 (3.5%)	50 (11.6%)
Bethesda category 4 (FN or SFN)	230 (10.9%)	69 (6.6%)	47 (7.1%)	39 (1.4%)	11 (0.6%)	7 (1.6%)
Bethesda category 5 (Suspicious for malignancy)	42 (2.0%)	0 (0)	23 (3.5%)	136 (5.0%)	1 (0.1%)	101 (23.5%)
Bethesda category 6 (Malignancy)	501 (23.7%)	0 (0)	501 (75.9%)	319 (11.8%)	2 (0.1%)	239 (55.6%)

Data are number of nodules. Percentages do not add up to 100% because of rounding.

AUS = atypia of undetermined significance, FLUS = follicular lesion of undetermined significance, FN = follicular neoplasm, SFN = suspicious for a follicular neoplasm.

**Table 3:** Outcomes of the Pooled and Matched Cohorts.

Outcomes	Pooled Cohorts			Propensity Score Matching			Inverse Probability Weighting		
	CNB (n = 2,114)	FNA (n = 2,708)	P value	CNB (n = 1,615)	FNA (n = 1,615)	P value	CNB (n = 2,114)	FNA (n = 2,708)	P value
Non-diagnostic results rate	5.2%	12.1%	<0.001	5.7%	11.4%	<0.001	5.7%	13.8%	<0.001
Malignancy rate	23.7%	11.8%	<0.001	22.5%	14.7%	<0.001	22.7%	9.0%	<0.001
Complication rate	0.33% (7 of 2,114)	0.074% (2 of 2,708)	0.048	0.25%	0.06%	0.375	0.23%	0.13%	0.188

Final diagnoses were obtained for 1,703 of 2,114 nodules (80.6%) in the CNB group and 2,190 of 2,708 nodules (80.9%) in the FNA group and they were included in evaluating diagnostic performance. Six hundred forty five of 1,703 nodules (37.9%) in the CNB group and 461 of 2,190 nodules (21.1%) in the FNA group were diagnosed after surgery. The sensitivity for diagnosing malignancy in the CNB group (75.9%; 501 of 660) was significantly higher than in the FNA group (55.6%; 239 of 439; *p* < 0.001). However, the specificities were similar (100%, 1,043 of 1,043, and 99.9%, 1,758 of 1,760, respectively). In terms of false negative rates, both CNB (2.4%; 21 of 883) and FNA (1.0%; 17 of 1,629) showed low false negative.

Propensity score matching and inverse probability weighting showed unchanged results in non-diagnostic result rate, malignancy rate, sensitivity, and specificity. Although there was a significant difference between the CNB and FNA groups in terms of the complications rate in unmatched cohorts (*p* = 0.048), there was no significant difference following propensity score matching and inverse probability weighting (*p* = 0.375, *p* = 0.188, respectively).

### Subgroup analysis according to the nodule size ≥ 1 cm

The baseline characteristics of the study patients according to the nodule size ≥ 1 cm are shown in Supplementary Table 1. In matched cohorts, there were no significant difference between the CNB group and the FNA group for any covariate. The results of the CNB and FNA groups and their final diagnoses are summarized in Supplementary Table 2. Non-diagnostic result rate was consistently lower in the CNB group (4.7%; 65 of 1,396 nodules) than in the FNA group (11.0%; 162 of 1,475 nodules; *p* < 0.001) (Supplementary Table 3). Malignancy rate was significantly higher in the CNB group (13.9%; 194 of 1,396 nodules) than in the FNA group (7.2%; 106 of 1,475 nodules; *p* < 0.001). Inverse probability weighting was unchanged for the malignancy rate, with propensity score matching indicating no significant difference between the CNB and FNA groups (*p* = 0.330). There was no significant difference between the CNB (0.36%; 5 of 1,396) and FNA groups (0.068%; 1 of 1,475) with respect to the complication rate (*p* = 0.115) (Supplementary Table 3). The sensitivities (60.8%, 194 of 319, vs. 60.9%, 81 of 133, respectively) and specificities (100%, 768 of 768, vs. 99.9%, 1,098 of 1,099, respectively) were consistently similar between two groups.

## Discussion

In our study, we compared the outcomes of CNB and FNA in a large population of patients with initially detected thyroid nodules by performing a propensity score analysis. Our observational study showed that CNB achieved a significantly lower non-diagnostic result rate and higher malignancy rate than that of FNA. These results were consistently evident across propensity score matching, inverse probability weighting, and subgroup analysis. Although there was a significant difference in complications rate between the two groups in unmatched cohorts, there was no significant difference in matched cohorts. The sensitivity of the CNB group was significantly higher than that of the FNA group; however, the specificities were similar. Subgroup analyses according to the nodule sizes ≥ 1 cm also showed consistent results. Therefore, CNB appears to be a promising and safe diagnostic tool for patients with initially detected thyroid nodules. Currently, there are no definitive guidelines on how CNB should be used in the evaluation of initially detected thyroid nodules. Although there were limited data on CNB, we hope that consistent evidence favoring the use of CNB including the results of our study will be validated by future clinical trials.

There were significant differences between the two groups in terms of nodule size, nodule composition, margin, echogenicity, and calcification in pooled cohorts. Similarly, higher malignancy rates were evident in the CNB group relative to that of the FNA group (23.7% vs. 11.8%, respectively). This was probably due to the significantly larger nodule sizes evident in the CNB group (1.68 cm) relative to that of the FNA group (1.24 cm), with the incidence of suspicious US characteristics being significantly higher in the CNB group than in the FNA group. To reduce the effects of selection bias and potential confounding variables in this observational study, propensity score matching and inverse probability weighting were performed. In matched cohorts, significant differences between the CNB group and the FNA group for all covariates were abolished. The malignancy rate was consistently higher in the CNB group than in the FNA group via validation by propensity score matching (22.5% vs. 14.7%, respectively) and inverse probability weighting (22.7% vs. 9.0%, respectively).

Although a small population pilot study (31 patients) reported that CNB was more effective for initially detected suspicious thyroid nodules compared to FNA^18^, this large population study verified its efficacy and safety. Current literature has demonstrated that CNB significantly lowered the pooled proportion of non-diagnostic results, higher sensitivity for diagnosing malignancy when compared against FNA for nodules with previous non-diagnostic FNA results^10, 11, 15^ and previous indeterminate FNA results^14, 15^. The advantages of CNB may be explained due to its ability to sample large amounts of tissue, assess histologic architecture (rather than cytological evaluation) and function on a low rate of operator dependence, if targeting of thyroid nodules is successful^15^. In terms of cost, a recent study demonstrated that CNB can detect the benign thyroid nodules that are classified as previous indeterminate FNA results, and these patients can avoid diagnostic surgery and hospitals can reduce their surgical costs by one-third^33^. There is no study comparing the costs of CNB and FNA and further study will be needed.

In our study, there were no major complications after CNB or FNA. Minor complications were rare in both CNB and FNA. Complications were observed in only 7 cases of the 2,114 patients after CNB (0.33%) and in only 2 cases of the 2,708 patients after FNA (0.074%). Before 1980, large-needle biopsy (14-gauge) without US guidance was less effective and yielded more complications^34, 35^; however, modern, spring-activated biopsy needles (18–22 gauge), under US guidance, can achieve lower complication rates. These findings suggest that both CNB and FNA procedures are safe, well tolerated, and have a low incidence of complications. Nevertheless, operator experience is important for performing CNB to minimize procedure-related complications. Therefore, CNB should only be performed by clinically experienced and trained operators under US guidance, who are familiar with the US anatomy of the thyroid and perithyroidal areas^36^.

Since thyroid malignancies smaller than 1 cm are often considered clinically insignificant, the recent American Thyroid Association guidelines have recommended that only nodules > 1 cm should be evaluated^1^. Therefore, we performed subgroup analyses accordingly to nodule sizes ≥ 1 cm, thereby showing consistently significantly lower non-diagnostic result rates and higher malignancy rates evident in the CNB group relative to that of the FNA group, in pooled and matched cohorts alike. In addition, there was no significant difference between the CNB and the FNA groups in terms of complication rate and diagnostic accuracy. These findings affirm CNB efficacy for nodule sizes ≥ 1 cm.

The major limitation of our study was that it was based on observational data. Our study results should be interpreted with some reservation because of the possibility of a selection bias towards suspicious nodules owing to US usage for the CNB group. Secondly, as this was a single-center study, the generalizations of these results may be limited. Third, efficacy of CNB versus FNA may not be an accurate comparison even with propensity score matching and inverse probability weighting when bias is at the level of the ordering physician.

In conclusion, this study demonstrated that the observations of significantly lower non-diagnostic result rates and higher malignancy rates in the CNB group compared to the FNA group were overall consistent in propensity score and subgroup analyses, therefore, CNB appears to be a promising and safe diagnostic tool for patients with initially detected thyroid nodules.

## Electronic supplementary material

Supplementary Dataset 1
