# Supplementary material for: Efficacy and safety of core-needle biopsy in initially detected thyroid nodules via propensity score analysis
Source: Sci Rep. 2017 Aug 15;7:8242. doi: 10.1038/s41598-017-07924-z (PMC5557918; doi:10.1038/s41598-017-07924-z)
Supplement: Supplementary file 1 — Supplementary Dataset 1 [file 41598_2017_7924_MOESM1_ESM.doc]

**Efficacy and safety of core-needle biopsy in initially detected thyroid nodules via propensity score analysis**

Chong Hyun Suh, MD^1, 2^, Jung Hwan Baek, MD, PhD^1^, Young Jun Choi, MD^1^, Tae Yong Kim, MD, PhD^3^, Tae Yon Sung, MD^4^, Dong Eun Song, MD, PhD^5^, Jeong Hyun Lee, MD, PhD^1^

^1^Department of Radiology and Research Institute of Radiology, University of Ulsan College of Medicine, Asan Medical Center, 86 Asanbyeongwon-Gil, Songpa-Gu, Seoul 138-736, Republic of Korea

^2^Department of Radiology, Namwon Medical Center, 365, Chungjeong-Ro, Namwon-Si, Jeollabuk-Do 590-702, Republic of Korea

^3^Department of Endocrinology and Metabolism, University of Ulsan College of Medicine, Asan Medical Center, 86 Asanbyeongwon-Gil, Songpa-Gu, Seoul 138-736, Republic of Korea

^4^Department of Surgery, University of Ulsan College of Medicine, Asan Medical Center, 86 Asanbyeongwon-Gil, Songpa-Gu, Seoul 138-736, Republic of Korea

^5^Department of Pathology, University of Ulsan College of Medicine, Asan Medical Center, 86 Asanbyeongwon-Gil, Songpa-Gu, Seoul 138-736, Republic of Korea

Supplementary Materials

*Histopathologic Analysis of CNB specimens and Cytopathologic Analysis of FNA*

Bethesda category 1 (non-diagnostic) includes: (1) the absence of any identifiable follicular thyroid tissue; (2) presence of follicular cells that are regarded as normal thyroid tissue; and (3) presence of tissue containing only few follicular cells insufficient for diagnosis. Bethesda category 2 (benign) includes colloid nodules, nodular hyperplasia, and lymphocytic thyroiditis. Bethesda category 3 (atypia of undetermined significance and follicular lesion of undetermined significance) include nodules characterized by the presence of atypical cells that are non-diagnostic of “suspicious malignancy” or “malignancy”; in addition to including cellular follicular nodules that do not fulfill the criteria for “follicular neoplasm/suspicious for follicular neoplasm. Nodules with histologic features favoring follicular neoplasm were categorized as Bethesda category 4 (follicular neoplasm or suspicious for a follicular neoplasm). Nodules were categorized as Bethesda category 5 (suspicious for malignancy) if the specimen exhibited atypia with insufficient evidence for a definitive diagnosis of malignancy. Specimens exhibiting unequivocal features of cancer were categorized as Bethesda category 6 (malignancy).

Supplementary Table 1. Baseline Characteristics of the Pooled and Matched Cohorts (nodule size ≥ 1cm)

|  | Pooled Cohorts | | | |  | Matched Cohorts | | | |
| --- | --- | --- | --- | --- | --- | --- | --- | --- | --- |
| Characteristics | CNB  (n = 1,396) | FNA  (n = 1,475) | P value | Standardized Difference |  | CNB  (n = 990) | FNA  (n = 972) | P value | Standardized Difference |
| Clinical factors |  |  |  |  |  |  |  |  |  |
| Age (years) | 54.0 ± 13.0 | 54.2 ± 12.4 | 0.657 | 0.0398 |  | 53.9 (12.7%) | 53.3 (12.7%) | 0.332 | -0.0438 |
| Sex (M:F) | 302:1,094 | 351:1,124 | 0.168 | 0.06 |  | 214:776 | 201:771 | 0.611 | -0.023 |
| US Characteristics |  |  |  |  |  |  |  |  |  |
| Nodule size (cm) | 2.22 ± 1.19 | 1.72 ± 0.84 | < 0.001 | -0.4293 |  | 1.95 ± 0.92 | 1.87 ± 0.93 | 0.059 | -0.0852 |
| Composition, n (%) |  |  | < 0.001 | 0.3108 |  |  |  | 0.170 | 0.1014 |
| Solid | 1,035 (74.1%) | 870 (59.0%) |  |  |  | 684 (69.1%) | 710 (73.1%) |  |  |
| Predominantly solid | 285 (20.4%) | 398 (27.0%) |  |  |  | 238 (24.0%) | 193 (19.9%) |  |  |
| Predominantly cystic | 69 (4.9%) | 177 (12.0%) |  |  |  | 61 (6.2%) | 62 (6.4%) |  |  |
| Cystic | 7 (0.5%) | 30 (2.0%) |  |  |  | 7 (0.7%) | 7 (0.7%) |  |  |
| Shape, n (%) |  |  | 0.051 | 0.035 |  |  |  | 0.880 | 0.0229 |
| Ovoid to round | 1,231 (88.2%) | 1,333 (90.4%) |  |  |  | 885 (83.4%) | 865 (89.0%) |  |  |
| Taller than wide | 37 (2.7%) | 22 (1.5%) |  |  |  | 21 (2.1%) | 19 (2.0%) |  |  |
| Irregular | 128 (9.2%) | 120 (8.1%) |  |  |  | 84 (8.5%) | 88 (9.1%) |  |  |
| Margin, n (%) |  |  | < 0.001 | 0.1773 |  |  |  | 0.577 | 0.0474 |
| Smooth | 863 (61.8%) | 1,032 (70.0%) |  |  |  | 634 (64.0%) | 635 (65.3%) |  |  |
| Spiculated | 77 (5.5%) | 43 (2.9%) |  |  |  | 35 (3.5%) | 40 4.1%) |  |  |
| Ill-defined | 456 (32.7%) | 400 (27.1%) |  |  |  | 321 (32.4%) | 297 (30.6%) |  |  |
| Echogenicity, n (%) |  |  | < 0.001 | 0.2801 |  |  |  | 0.390 | 0.0784 |
| Isoechoic | 831 (59.5%) | 857 (58.1%) |  |  |  | 53 (5.4%) | 68 (7.0%) |  |  |
| Hypoechoic | 437 (31.3%) | 523 (35.5%) |  |  |  | 336 (33.9%) | 310 (31.9%) |  |  |
| Markedly hypoechoic | 119 (8.5%) | 70 (4.7%) |  |  |  | 592 (59.8%) | 587 (60.4%) |  |  |
| Hyperechoic | 9 (0.6%) | 25 (1.7%) |  |  |  | 9 (0.9%) | 7 (0.7%) |  |  |
| Calcifications, n (%) |  |  | < 0.001 | 0.4002 |  |  |  | 0.608 | 0.0612 |
| None | 996 (71.3%) | 1,269 (86.0%) |  |  |  | 64 (6.5%) | 58 (6.0%) |  |  |
| Microcalcifications | 99 (7.1%) | 65 (4.4%) |  |  |  | 106 (10.7%) | 120 (12.4%) |  |  |
| Macrocalcifications | 235 (16.8%) | 121 (8.2%) |  |  |  | 25 (2.5%) | 20 (2.1%) |  |  |
| Rim calcifications | 66 (4.7%) | 20 (1.4%) |  |  |  | 795 (80.3%) | 774 (79.6%) |  |  |

Supplementary Table 2. CNB and FNA results and final diagnosis for initially detected thyroid nodules (nodule size ≥ 1cm)

|  | Total CNB  (n = 1,396) | Final diagnosis (n = 1,087) | | Total FNA  (n = 1,475) | Final diagnosis (n = 1,232) | |
| --- | --- | --- | --- | --- | --- | --- |
|  |  | Benign  (n = 768) | Malignant  (n = 319) |  | Benign  (n = 1,099) | Malignant  (n = 133) |
| Bethesda category 1 (Non-diagnostic) | 65 (4.7%) | 20 (2.6%) | 2 (0.6%) | 162 (11.0%) | 42 (3.8%) | 5 (3.8%) |
| Bethesda category 2 (Benign) | 637 (45.6%) | 621 (80.9%) | 16 (5.0%) | 1,008 (68.3%) | 1,002 (91.2%) | 6 (4.5%) |
| Bethesda category 3 (AUS or FLUS) | 272 (19.5%) | 61 (7.9%) | 53 (16.6%) | 90 (6.1%) | 30 (2.7%) | 16 (12.1%) |
| Bethesda category 4 (FN or SFN) | 211 (15.1%) | 66 (8.6%) | 44 (13.8%) | 80 (5.4%) | 24 (2.2%) | 5 (3.8%) |
| Bethesda category 5 (Suspicious for malignancy) | 17 (1.2%) | 0 (0) | 10 (3.1%) | 29 (2.0%) | 0 (0) | 20 (15.0%) |
| Bethesda category 6 (Malignancy) | 194 (13.9%) | 0 (0) | 194 (60.8%) | 106 (7.2%) | 1 (0.1%) | 81 (60.9%) |

Data are number of nodules. Percentages do not add up to 100% because of rounding.

AUS = atypia of undetermined significance, FLUS = follicular lesion of undetermined significance, FN = follicular neoplasm, SFN = suspicious for a follicular neoplasm.

Supplementary Table 3. Outcomes of the Pooled and Matched Cohorts (nodule size ≥ 1cm)

|  | Pooled Cohorts | | | Propensity Score Matching | | | Inverse Probability Weighting | | |
| --- | --- | --- | --- | --- | --- | --- | --- | --- | --- |
| Outcomes | CNB  (n = 1,396) | FNA  (n = 1,475) | P value | CNB  (n = 990) | FNA  (n = 972) | P value | CNB  (n =1,396) | FNA  (n = 1,475) | P value |
| Non-diagnostic results rate | 4.7% | 11.0% | < 0.001 | 5.3% | 9.9% | < 0.001 | 5.2% | 13.4% | < 0.001 |
| Malignancy rate | 13.9% | 7.2% | < 0.001 | 11.0% | 9.7% | 0.330 | 12.4% | 4.7% | < 0.001 |
| Complication rate | 0.36%  (5 of 1,396) | 0.068%  (1 of 1,475) | 0.115 | 0.2% | 0.1% | - | 0.27% | 0.04% | 0.007 |
